# The role of information in consumer preferences for sustainable certified palm oil products in Germany

**DOI:** 10.1371/journal.pone.0271198

**Published:** 2022-07-25

**Authors:** Christoph Richartz, Awudu Abdulai

**Affiliations:** Department of Food Economics and Food Policy, Institute of Food Economics and Consumption Studies, Kiel University, Kiel, Germany; University of Florida, UNITED STATES

## Abstract

Food products are often subject to information asymmetries, which are commonly supposed to be reduced by labels and certifications. However, as the number of labels increases, consumers tend to get confused, bored or impatient and stop using them to make product choices. This study uses data from a discrete choice experiment, conducted in Germany, to analyze consumers’ preferences and willingness-to-pay (WTP) for sustainability indicators on products that contain palm oil as an ingredient. Since information is crucial to the assessment and awareness of, or attendance to, labels on consumer products, this study assesses the effect of factual information on preferences as well as attribute-processing strategies. We use a hybrid latent variable model that allows us to jointly examine the response to the stated choice component and the responses to attribute processing questions, thus capturing attribute non-attendance (ANA) to specific labels while controlling for heterogenous preferences. Our results reveal that the attribute ‘organic’ receives the highest monetary valuation in the overall sample as well as in the information intervention, and the no-information intervention groups. The results also show that providing additional information tends to change consumers’ non-attendance patterns as well as WTP values. In particular, the information intervention tends to increase consumers’ WTP and decreases their ANA for sustainability-indicating attributes. The findings suggest that the attribute ‘organic’ has the potential to be ranked highest across the entire latent variable structure, making it the most promising attribute for promoting sustainable palm oil use across consumer groups.

## Introduction

Consumers normally face uncertainties, because various food product attributes are incomprehensible or intransparent credence type attributes. Credence characteristics are product characteristics for which utility cannot be exactly determined even after consumption [1]. Examples are sustainability properties indicated by sellers, which buyers cannot verify without very high pre- and post-buying costs. Information asymmetry can therefore lead to market failure, as the desire to match food choices with personal preferences may fail due to lack of information on the consumer side [2]. Since consumers need reliable information to help them in their consumption decisions to maximize utility, specific labels and certifications are normally used to provide effective information to them. The recent empirical literature shows that the concept of signaling tend to motivate consumers to increase their willingness-to-pay (WTP) for food products. For example, Van Loo et al. [3] and [4] find increased WTP values or strong consumer preferences for certain quality indicating meat product attributes. Some studies also find increased WTP for fair trade and organic quality and other environmental sustainability indicators for regional agricultural products, as well as for products from tropical regions [e.g. 5–8].

The recent literature in the field of consumer preferences for palm oil suggests that consumers prefer certification measures that clearly reduce transparency problems within the supply chain [9]. Gasslers and Spillers’ [9] findings reveal that consumers are willing to pay a price premium of €0.85 for a Roundtable on Sustainable Palm Oil (RSPO) certified chocolate product. Results by Hinkes and Christoph-Schulz [10] indicate that consumers on average prefer palm-oil-free products over certified products. The authors explain this choice behavior partly as a result of consumers’ health concerns and a potential lack of trust in certification [10]. However, even though the authors of this study have integrated more labels into their choice tasks, they still neglect the fact that people do or do not pay specific attention to some product attributes. In other words, that people rely on heuristics. Not accounting for such heuristics may lead to biased WTP estimates in the latent class model presented in Hinkes and Christoph-Schulz [10]. Our study extends the approaches used in previous studies and attempts to shed more light on precisely these behavioral structures, as multiple labels in the market can also become a problem for consumers. Since consumers are often faced with numerous labels, the notion of ‘bounded rationality’ [11] raises the question of how and whether labels still facilitate consumers’ product choices today.

Some studies have revealed that as the number of labels increases, consumers tend to get confused, bored or impatient, with the probability of their ability to understand the individual labels and their informative value decreasing [2, 12]. As a consequence, they no longer rely on labels when making choices or buying products. The use of heuristics refers to the application of simplistic decision-making rules, which may lead to biased estimates when not considered in statistical approaches [13]. Consumers tend to use heuristics when situations are too complex to assess, when they are uncertain, or when motivation and potential personal consequences are low [14] This simplification can, for example, result in the non-attendance to certain attributes when choosing products, the so-called attribute non-attendance (ANA). We often find this concept in choices of low-involvement goods such as food products [15], where consumers use routine purchasing patterns or heuristics that is, deliberately leaving out some attributes when evaluating products. As indicated above, recent literature shows that taking into account ANA sheds a more profound light on consumers’ decision-making behavior and that ignoring these heuristics can bias empirical estimates. More specifically, studies show changing WTP values for sustainability indicators on food products when ANA is included in the estimates [e.g. 16–18]. Since the consideration of sustainability indicators in food choice situations is essential for products’ success, and thus the achievement of sustainability goals, it is necessary to examine whether and how ANA can be influenced, for example, through public information campaigns or by information imparted at the point of sale. Our study attempts to fill this gap in the literature by addressing this issue.

We use a discrete choice experiment to determine consumer preferences for certified palm oil, and explore the effect of factual information on consumers’ food choice behavior and ANA patterns. Our study contributes to the literature by using a hybrid latent variable model that allows us to jointly examine the response to the stated choice component and to attribute-processing questions, thus also capturing stated ANA [16]. The objective of this approach is to identify consumer preferences and WTP for sustainability indicators and to identify which labeling institution is best suited to promoting sustainable palm oil consumption, as consumers pay the most attention to a specific certification when making product choices.

As part of the present study, an information treatment group received factual information on the topic of palm oil cultivation before continuing with an unlabeled Choice Experiment (CE), in which hypothetical purchasing decisions had to be made between palm oil-containing product alternatives. For example, respondents received information about global problems resulting from palm oil cultivation, information on yields or employment and livelihood opportunities for farmers in cultivation regions. Since our focus is on labels relating to environmental protection, no health claims were included in the information treatment. No organizations, certification bodies or other institutions were presented negatively or positively in this context, in order to prevent a biased attitude being formed by participants towards certain labels or regulatory mechanisms. This distinguishes our work from other studies, that often provide little information about the controversy of the topic or which strictly provide information about the labels themselves, such as the meaning of the label or the criteria of production conditions. One informational side (positive / negative) is often neglected, so that it can be assumed that respondents conduct the experiments in a biased manner [9, 10, 19]. We argue at this point that the linking of factual information that is, the fact that people have or do not have knowledge about certain topics is more in line with reality, and that consumers evaluate facts and thus products and labels with the help of their personal knowledge. This would be contrasted by a consumer who is strictly informed about certain labels. However, it is unclear how consumers evaluate products using a label without having sufficient background knowledge. Here one can assume two scenarios. Either the person must have received the corresponding factual information in addition to the information about the label to be able to assess the product or he or she evaluates a label without knowing the background exactly. In the latter case, however, he or she would not know whether the label is even reasonable or trustworthy or not and the decision would be made on the basis of more, though not necessarily processable, information. We argue that providing information only about the label itself would not solve the previously mentioned problem of information overload.

When consumers are faced with product choice under many different labels, all of which they understand the meaning of, they would still have to decide which institution or label they trust the most to make a final product choice. However, it is unrealistic to expect every consumer to learn everything about every label. In contrast, it seems more realistic for the industry or official regulatory bodies to focus on labels that are understood, trusted and accepted by the consumer. Therefore, it is not only essential to determine preferences and WTP for product attributes, but it is just as important to understand which of these attributes are being paid attention to in the first place. Thus, we also contribute to scholarly knowledge by using the analysis of consumers’ attribute-processing strategies, to identify the label to which consumers pay most attention to. Further, we contribute to the literature by presenting, to the best of our knowledge, the first study to examine the effect of factual information on ANA patterns related to attributes of food products. Finally, this study contributes to the literature by providing insight into both attitudes and expectations of palm oil using companies in the food sector and expectations of consumers through the use of primary data.

The rest of the paper is structured as follows. The next section discusses the background of oil palm cultivation and palm oil in food products, as well as the literature relating to this issue. Section three outlines the econometric framework used in the analysis, while section four presents the survey design. The empirical results are discussed in section five, and the final section presents conclusions and implications.

### The market of sustainable palm oil

In 2015, the United Nations added sustainable consumption and production to the agenda of the Sustainable Development Goals (SDG). In particular, goal 12 of the SDG aims to reduce future economic, environmental and social costs, strengthen economic competitiveness, increase incomes, reduce poverty and improve food security. The goal is to improve people’s quality of life worldwide by reducing resource consumption, degradation and environmental pollution [20]. Since these commitments affect global supply chains, action by both industrialized and developing countries is required. For consumers of products with ingredients from many parts of the world, it is therefore of paramount importance to be able to distinguish between more or less sustainable products and production processes. This requires consumer knowledge and education, i.e., information about sustainable consumption as well as interrelationships and consequences on a global scale. Knowledge and education are indispensable prerequisites for promoting sustainable development and improving people’s ability to address environmental issues. They are necessities for bringing about a change in people’s consciousness so that they are able to assess and address their concerns about sustainable development. They are also crucial for creating environmental and ethical awareness, as well as values and behaviors compatible with sustainable development, and for effective public participation in decision-making [e.g. 21, 22].

The present study focuses on products that contain palm oil. Due to its prominent role in the world market for vegetable oils and with its global supply chains, palm oil is of particular importance. With a production volume of about 75.45 million metric tons, palm oil is one of the most produced, consumed, and globally traded vegetable oils. The world’s major palm oil producers are Indonesia and Malaysia with 58% and 26% of global production, respectively. The main importers are India (17%), China (14%) and the European Union (14%) [23]. Compared to other oil crops, oil palm is the most productive, with a current global average yield of around 3.5 tons per hectare and maximum yields of up to 6 tons per hectare [24, 25]. The oil palm is a perennial, evergreen tree, cultivated in equatorial areas rich in biodiversity [25], and the cultivation is mostly done as a monocrop for commercial purposes, with some exceptions in West and Central Africa, where it can also be found as part of agroforest systems and more often used for domestic consumption [24, 26]. In this study, we focus on the German market, where it is estimated that 48% of all palm oil imports are not certified. Certified oil is divided into different types, such as *segregated* (27%), *mass balance* (21%), or *organic* (1%) oil. About 85% of the palm oil in food products is certified (i.e. *segregated*, *mass balance*, *book and claim*, and others) and 5% *organic* certified [27]. Segregated refers to palm oil from different certified sources and is kept separate from ordinary palm oil throughout the supply chain. The category ‘mass balance’ implies that millers, refiners or retailers can acquire palm oil from both certified and non-certified sources and process them together, without the need for separate supply chains. The Roundtable on Sustainable Palm Oil (RSPO) ‘book and claim’ describes a system where the supply chain is not monitored for the actual presence of sustainable palm oil. Stakeholders in the supply chain are allowed to buy RSPO credits [28].

Negative impacts arising from palm oil production such as deforestation, habitat loss, decline in species populations and biodiversity have been very well documented [29–32]. Additionally, if slash and burn practices are used to clear the land for cultivation, smoke and toxic compounds with negative impacts on people and wildlife can occur [33, 34]. Other indirect effects are downstream water pollution from fertilizers or negative impacts on soils and waterbodies due to toxic compound-releasing palm oil mill effluents [35, 36]. However, manual weeding of plantations and harvesting of fresh fruit bunches is labor intensive, which makes cultivation an important livelihood option in growing regions [24]. Moreover, due to its high yields per hectare, the oil palm has the potential to meet a large part of the global demand for vegetable oils with relatively low land use. These conflicting positions, on the one hand the resulting social and environmental problems, and on the other hand, the potential sustainability of the commodity, underline the importance for reliable guidelines governing sustainable consumer behavior. This importance is exacerbated by the fact that palm oil is found in many products, which increases the demand for crude palm oil and thus the area under cultivation. It is estimated that close to 50% of products found in supermarkets contain palm oil, highlighting the importance of this commodity in terms of sustainable consumer choices [37]. However, European consumers have become increasingly concerned about the adverse effects of palm oil production. Reactions to its use have been, for example, boycotts or campaigns led by non-governmental organizations (NGO) against plantation-funding institutions, retailers and processors [38].

To change consumption patterns and preferences in regions with high demand, such as the EU, consumers need reliable sources of information to address the problems associated with palm oil production. Although the RSPO aims to set a global sustainability standard for palm oil production, there are various uncertainties about this body. Founded in 2004, the RSPO is a voluntary membership association bringing together different private stakeholders, such as investors, processing firms, growers and NGOs, to promote economic, social and environmental sustainability in the production and use of palm oil. However, it has been referred to as pro-industry biased [39], and criticized for not addressing the real problems of unsustainable cultivation [37, 40]. The main reasons cited for this assessment include, for example, weak reporting, monitoring and enforcement mechanisms in the context of corporate sustainability or the lack of an effective system for monitoring and enforcing compliance at the micro level [39, 41]. Furthermore, there are different categories within the RSPO certification pathway such as ‘mass balance’ or ‘book and claim’. For example, regarding the certification category ‘mass balance’, the RSPO states on its official website that a product can be RSPO-certified under the mass balance system and thus carry a label. Sustainable palm oil from certified sources can be physically included in the product, but may be mixed with conventional palm oil. In fact, in this case, the consumer does not have a full guarantee that the product actually contains sustainable palm oil [42]. However, recent literature shows that consumers prefer segregated supply chains and uncertainty-reducing measures [9]. Therefore, the certification structure may create transparency problems for less informed market participants. Nevertheless, this system also has advantages, as producers are not required to maintain separate supply chains, which can reduce transaction costs and allow for a gradual shift to more sustainable supply chains [9]. Another issue of great concern is the fact that many consumers are relatively unaware of the label. This raises the question of whether RSPO indeed represents an effective approach to sustainable oil palm cultivation. Thus, even though palm oil is no longer hidden in ingredient lists of food products [43], considerable communication efforts will be required to increase consumer awareness and demand for sustainable palm oil [9, 39]. As indicated earlier, studies have shown an increased WTP or preferences for certified products. It can be concluded that clear preference structures with respect to particular certification bodies are generally not given across consumer groups or product types. Producers face the challenge of making the (right) decision for or against the use of certain labels, since investing in certification must be profitable and there are considerable differences regarding trust and acceptance of labels. Therefore, the next section focuses on the palm oil sector in Germany through the use of primary data. Both shared and conflicting opinions could provide stakeholders with important insights on how to maximize the utility of all market participants. We want to emphasize that only by identifying the most appropriate or accepted institution can an economically, environmentally and socially precise instrument for promoting sustainable consumption be developed and implemented efficiently.

In addition to consumer data, we collected data from companies in Germany that use palm oil to manufacture their confectionary and/ or chocolate products. The short survey was carried out in February 2020. About 87 (74%) companies that participated in our survey belong to the category of palm oil users. Table A1 in S1 Appendix shows an overview of the company sizes we surveyed. Within the category of oil palm users, only 4.6% stated that another agricultural commodity could *easily* serve as a substitute in their products, while 9.2% indicate that they could switch to another commodity with *little expenses*.

About 45% of the respondents indicated that palm oil can be replaced at *very high costs*, while 30% stated that it *cannot be replaced at all*. On the use of palm oil, 84% of the companies reported that they *exclusively* use certified oil, while 11% stated that they *mostly* use it. However, the certified commodities used by the companies differ. For example, 63% use RSPO-certified palm oil with segregated supply chains, 22% use palm oil from sources with other RSPO certification standards, such as book and claim. Organic certified palm oil is *sometimes* used by 11% of the companies. When asked whether the companies can imagine using only organic palm oil in the future, their responses differ, with 46% indicating that it is *likely* or *very likely*, while 54% stated that it is *unlikely* or *very unlikely*. 44% of the producers stated that they do not have any information on the region of origin of the commodity used for their products. When companies were asked about their future expectations of developments in the market and of sustainability aspects with regard to their own ability to adapt to more sustainable production methods, divided opinions also emerge. About 60% have *positive* or *extremely positive* expectations of the aforementioned, prospective transformations, while 40% of respondents already have concrete plans to adapt their company to more sustainable concepts and production methods. Nevertheless, the companies explained that they had used little or no organic palm oil so far due to high costs (53%), availability (19%) and uncertainties regarding secure supply chains (9%). However, most companies (67%) cannot imagine implementing a project initiative of their own, such as those that exist in West Africa, to ensure that their production requirements are met by means of a sustainable palm oil supply chain. Interestingly, 28% of the companies indicated that they could imagine participating in the implementation of a sustainable palm oil value chain project.

### Econometric framework

Our empirical analysis employs the framework by Hess and Hensher [44], where the link between the stated choice and the attribute-processing component in the model is made by a latent variable (LV) which relates to the unobserved respondent-specific importance measure for each attribute. Starting from the perspective of the traditional random utility model, we define the utility of alternative *i* for respondent *n* in choice scenario *t* as *U*_*int*_ = *V*_*int*_+*ε*_*int*_. With *J* alternatives (*j* = 1, …, *J*), the probability of alternative *i* being chosen is given by:

$$P_{int}=P(V_{int}+\varepsilon_{int}>V_{\mathrm{jnt}}+\varepsilon_{jnt},\;\forall j\neq i)$$ (1)

where *V*_*int*_ represents the deterministic and *ε*_*int*_ the random component of the utility. The deterministic component of utility is given by a function of observed attributes *x* and estimated parameters *β*, *V*_*int*_ = *f*(*x*_*int*_, *β*), where a linear in parameters specification is employed. To account for preference heterogeneity, we employ a Mixed Multinomial Logit (MMNL) model, where we accommodate random variation across respondents in *β* with a type I extreme value distribution for the remaining error term ε. We define

$$P_{int}(\Omega )=\int_{\beta }\frac{e^{V_{int}(\beta )}}{\sum_{j=1}^{J}e^{V_{jnt}(\beta )}}h(\beta |\Omega )d\beta$$ (2)

where *β*~*h*(*β*|Ω), with Ω being a vector of parameters to be estimated. Since we work with repeated choice data, we follow Hess and Hensher [44] with the assumption of intra-respondent homogeneity so that the likelihood of the actual observed sequence of choices for respondent *n* is then given by:

$$L_{n}(\Omega )=\int_{\beta }[\prod_{t=1}^{T}P_{i^{*}nt}\;(\beta )]\;h(\beta |\Omega )d\beta$$ (3)

where i*nt refers to the alternative chosen by respondent *n* in choice situation *t*.

As indicated previously, in addition to capturing information on the choices, we also gathered information relating to respondents’ attribute-processing strategies. The approach employed in this study uses the mutually exclusive, stated attribute rankings to determine the attribute non-attendance (ANA) indicators. We treat the answers to information processing as dependent variables that are a function of the true underlying processing strategies. We assume that for every attribute *k*, each respondent *n* has an underlying, latent rating of attribute importance. This underlying, unobserved rating is thus given by a latent variable:

$$\alpha_{nk}=\varphi_{k}z_{n}+\sigma_{l}\;\eta_{nk}^{l}$$ (4)

where *z*_*n*_ represents respondents’ characteristics as well as answers to attitudinal questions relating to trust, consumption and environmental issues. $\eta_{nk}^{l}$ is a random term assumed to follow a standard normal distribution across respondents and across the *K* different attributes. The vector *φ*_*k*_ explains the effect of *z*_*n*_ on the latent variable *α*_*nk*_.

To model the probability for the response to the ranking question (ANA-indicators), we make use of a rank exploded MNL model, where the probability to fall between specific thresholds is influenced by the latent variable *α*_*nk*_. The mutually exclusive rankings for the *K* attributes are given by R_*k*_, *k* = 1, …, *K* where 1 ≤ R_*k*_ ≤ *K*, ∀*k*. We specify:

$$\gamma_{nk}=\zeta_{k}+\tau_{k}\alpha_{nk},\forall k$$ (5)

where *ζ*_1_ is set to 0 for normalization purposes. The conditional probability is then given as:

$$\upsilon_{nr}=\sum_{k=1}^{K}\delta_{\left(R_{k,r}\right)}\gamma_{nk},r=1,\ldots ,K$$ (6)

where $\delta_{\left(R_{k,r}\right)}$ is equal to 1 if R_*k*_ = *r*, i.e., if attribute *k* has ranking *r*, and 0 otherwise. With *ζ* and *τ* grouping together the individual elements *ζ*_*k*_ and *τ*_*k*_ respectively, the probability for the response to the ranking question is specified as:

$$L_{Rn}(\zeta ,\tau ,\alpha_{n})=\prod_{r=1}^{K-1}\frac{e^{\upsilon_{nr}}}{\sum_{s=r}^{K}e^{\upsilon_{ns}}}.$$ (7)

To link the latent variable *α*_*nk*_ which explains the answers to the non-attendance and ranking questions to the choice model, we use *α*_*nk*_ as shrinkage factors in the choice model component, thus allowing for a continuous measure of importance, i.e. using a latent variable scaling approach [16]. We replace the parameter *β*_*k*_ in the choice model component by $e^{\lambda_{k}\alpha_{nk}}$ by computing the attribute-specific scaling parameters *λ* = 〈*λ*_1_,…,*λ*_*K*_〉. We use two separate components to capture heterogeneity, *α*_*nk*_ and *β*_*k*_, to allow for the absence of a strict relationship between attribute importance and sensitivities, thus capturing any unrelated random heterogeneity in *β*_*k*_. Conditional on *α*_*nk*_ and *β*_*k*_ we specify:

$$P_{int}(\beta ,\lambda |\alpha_{n})=\frac{e^{\sum_{k=1}^{K}e^{\lambda_{k}\alpha_{nk}\beta_{k}x_{k,int}}}}{\sum_{j=1}^{J}e^{\sum_{k=1}^{K}e^{\lambda_{k}\alpha_{nk}\beta_{k}x_{k,int}}}}$$ (8)

where *x*_*k*,*int*_ is the *k*th component in *x*_*int*_. A positive estimate for *λ*_*k*_ here indicates that as the importance rating rises in value, so does the marginal sensitivity to attribute *x*_*k*_ [16]. Thus, the values of the attribute-processing component *L*_*Rn*_(*ζ*, *τ*, *α*_*n*_) are jointly modeled with the likelihood of the observed choice sequence *P*_*int*_(*β*, *λ*|*α*_*n*_).

Model- and group-specific WTP values for the different product attributes *X* are calculated as the rate of change in the attribute coefficient β divided by the rate of change of the price parameter *y*_*ps*_ (marginal rate of substitution). This is given as

$$WTP=-\left(\frac{\frac{\partial U}{\partial X}}{\frac{\partial U}{\partial P}}\right)=-\frac{\beta_{as}}{y_{ps}}$$ (9)

### Survey design and data description

To control for quantity effects, two different products, containing different amounts of palm oil were considered in the choice experiment (CE). In September 2019, we conducted face-to-face consumer pretests in front of a total of ten stores of the five leading German supermarket chains to determine relevant products as well as attributes of importance. Our pretest sample of 100 randomly selected respondents consisted of 52% women and had an average age of 41 years. The consumers were asked whether they regularly or at least occasionally buy the products of interest. They were then asked what attributes they pay most attention to when buying the products i.e., which attributes are crucial to their product choice. Finally, the interviewers recorded basic socioeconomic data as well as the location of the purchase and store type. Based on the results, we selected chocolate cookies and chocolate spread, two common food products where people pay attention to and value different attributes such as ingredients or certifications, as the products to analyze. Other products, such as margarine, were eliminated by the pretest because consumers revealed a pronounced indifference regarding product alternatives or attributes. In contrast to other studies, our pretest clearly indicates that consumers are mostly sensitive to the brand (36%) and chocolate type (28%) when buying chocolate cookies. In other choice experiments, however, the chocolate content of chocolate cookies was used as an attribute [10], which was perceived as marginally important in our pretest (5%). The pretest data on the chocolate spread results in an even clearer picture. 64% of the total sample based their shopping decision mainly on the brand. Therefore, we have included brand as an attribute in the CE in addition to sustainability indicators.

During the survey period from December 2019 to February 2020, we randomly sampled 460 respondents across Germany via postal contact. The personally addressed individuals were invited to participate in a survey about sustainability labels on food products. The postal invitation to the study featured different ways and possibilities for accessing the questionnaire. The questionnaire was then carried out on a browser-based platform. The participants were randomly sampled into the two product groups. The final distribution of evaluable questionnaires consists of 46% and 54% of participants in the cookie and the spread groups, respectively. Furthermore, 50% of the participants were randomly assigned to the information intervention group, while the rest did not receive any information intervention. We would like to emphasize again that the information provided refers exclusively to facts concerning oil palm cultivation and to advantages and disadvantages resulting from it (Section II in S1 Appendix). We also provided some very basic information on the displayed labels in both groups (Table A2 in S1 Appendix). Furthermore, our questionnaire contained general questions on socio-demographic characteristics, on attitudes towards environment and sustainability, shopping behavior, risk and trust attitudes, the choice experiment, and questions on price expectations and perceptions as well as attribute processing strategies and importance.

We use the RSPO label because it was developed to communicate sustainability in the palm oil sector. We also include the German organic label, as it is one of the well-known sustainability indicators in Germany and one of the most helpful labels from a consumer’s point of view [12]. Along with concerns about chemical substances, environmental ethics and protection are among the most important considerations consumers make when choosing organic products [45, 46] Due to the aforementioned potential effect of consumers being overwhelmed by too many sustainability labels and in addition to the RSPO and the German ‘Bio’ (organic) label, we have integrated a hypothetical sustainability label into the experiment in order to test acceptance via familiarity and easy interpretability. The appearance of the label is identical to the appearance of the ‘Haltungsform’ label, which provides consumers with information about animal husbandry and transparency indicators in the food retail sector in Germany. Using a simple, ascending system of four levels (1, 2, 3, 4), the label was developed to be as informative as possible, quickly and easily. A visualization can be found in Table A2 in S1 Appendix. The individual prices are calculated based on real prices across different supermarkets and quality categories. This means that we have included average prices from very low-priced private labels as well as average prices from the premium segment of products in the experiment. Based on our research, we decided on four price categories, as these realistically reflect the German market. We thus represent the lowest discount range, the more premium private label or low-priced brand range, the average brand range, and the average premium price range. The attribute brand simply describes whether or not the product is (in the participants’ view) a well-known brand. Table 1 presents the attributes and their corresponding levels.

**Table 1:** Attributes and attribute level in the choice experiments.

Attributes	
RSPO Label / Certification	No / Mass Balance / Segregated
German organic (Bio) Label / Certification	No / Yes
4-Level Label / Certification	No / Level 2 / Level 4
Brand	No / Yes
Price (spread / cookies)	1.29€, 1.99€, 2.79€, 3.99€ / 0.99€, 1.29€, 1.99€, 2.79€

Participants had to take part in the unlabeled CE that is, they had to choose one of the presented products in each of the six choice scenarios. A *no-buy* option was always selectable. Participants could therefore choose between three alternatives in each choice scenario. The labels were presented graphically, as shown in Table A2 in S1 Appendix. The participants were introduced to the hypothetical shopping scenario, by being asked to imagine that they had an intention to purchase and were faced with the occurring choice situation in the supermarket. An example of a choice set can be found in Table A3 in S1 Appendix. As in previous studies [47, 48], we decided to use a D-optimal design to reduce the number of choice tasks while guaranteeing a balanced design. The resulting generic choice sets were divided into three blocks. For further information on the very well documented development of choice experiments and experimental designs, we would like to refer the interested reader to e.g. [49, 50]. We could not determine strictly dominant alternatives and therefore did not remove any of the 18 choice situations from the experiment. The design was generated using Stata® 15.

Since it has been well documented in the literature [16, 51] that a hypothetical bias can be reduced using ex-ante hypothetical bias mitigation tools, and some studies reveal that cheap talk (CT) scripts work effectively in online surveys [52, 53], a CT script was implemented right before the actual CE was carried out. A translated version can be found in Section V in S1 Appendix.

Table 2 shows descriptive characteristics of our sample with comparative values of the German population. By comparing the descriptive values in Table 2, we find a relatively good match between our sample and the German population. For example, our dataset is characterized by 51% women, an average age of 46.5 years, a mean household income of €2000 - €3000, and an average household size of 2.35. However, there is a difference in the distribution with respect to university degrees. Since our postal distribution was randomized, it can be assumed that more highly educated population groups are more interested in participating in these kinds of study. As in Sandorf et al. [54], a quiz-score (presented in Table 2) captured knowledge levels regarding information on palm oil cultivation and discussions within both the intervention and the non-intervention groups. Participants indicated whether they agreed or disagreed with six problem statements, each of which had correct or incorrect solutions. Thus, a maximum score of 6 could be achieved. We found a significant difference in test scores between the intervention and the non-intervention group, with the former having a higher score. The questions from the knowledge quiz can be found in Section VI in S1 Appendix.

**Table 2:** Descriptive statistics: Sample compared to German population.

Dataset		Pooled Dataset	No Information Intervention	Information Intervention	Germany^2^
female		0.51	0.51	0.51	0.51
age		46.5	46.2	46.8	44.4
income category (€)	mean	2001–3000			2008–3399
	median	2501–3000			2000–2500
household-size	mean	2.35			2
	median	2			
no. children		1.42	1.2***	1.7***	1.16–2.0
education	lower education	0.39	0.38	0.39	0.35
	prof. education	0.30	0.32	0.28	0.48
	university degree	0.31	0.30	0.33	0.18
test score^1^		3.8	3.5***	4.0***	
region	Germany				
N		460	231	229	

***, **, * Significance of difference between groups at the 1%, 5%, and 10% levels, respectively.

^1^: Test score captured by knowledge quiz. The scale ranges from 0 (no correct answer), to a maximum of 6 (all questions answered correctly) points.

^2^: Sources: [55–63]

^3^: Prof. education refers to individuals who at least have completed vocational training under the dual system

As previously stated, one problem with the RSPO is that only few consumers are aware of the RSPO trademark, resulting in a weak market demand for it [39]. We can confirm this finding from our data. When asked “Are you familiar with the RSPO label?”, only 15% of participants in our sample answered in the affirmative. With regard to the German organic label, the situation is different. When asked “Are you familiar with the German Bio (organic) label?”, 65% of respondents answered “yes”. Images of the respective labels were attached to the questions. Further, we can confirm from our data that too many labels confront consumers with a situation of increased uncertainty [12]. When participants were presented with the statement “There are too may labels: The number of labels does not give me an effective basis for decision-making.”, most agreed with the statement by choosing *agree* (42%) or *strongly agree* (30%) on a Likert scale. Only 12% disagreed or strongly disagreed.

As previously mentioned, we also captured respondents’ price expectations prior to the CE. To elicit uncertainty in this consumer reference price, we used the questioning technique presented in Caputo et al. [64]. In addition, we formulated the questions in both a future-oriented and past-oriented way (Section VII in S1 Appendix). Table 3 presents summary statistics and mean comparisons of consumers’ reference prices by product and information status.

**Table 3:** Price expectation by product and information status.

	Mean Price expectation in €	Forward oriented in €	Backward oriented in €
Full Sample	2.73	2.73	2.74
Chocolate Cookies	2.41	2.39	2.44
Chocolate Spread	3.11	3.22	3.03
	**No-Information**	**Information**	
Full Sample	2.66*	2.81*	
Chocolate Cookies	2.35	2.48	
Chocolate Spread	3.02	3.20	

***, **, * Significance of difference between groups at the 1%, 5%, and 10% levels, respectively.

We find that consumers have a relatively accurate understanding of the prices of the products used in the experiment, which highlights the credibility of their subsequent product evaluation in the CE. Additionally, participants who received the information have significantly higher price expectations. Interestingly, a similar observation is made, even when the sample is split into product categories, although the result is not statistically significant.

The LV *α*_*nk*_ is described by each respondent’s characteristics as well as answers to attitudinal questions related to trust, consumption and environmental issues which are included in the model as *z*_*n*_ variables. Table 4 presents the variables used for our model specification in the LV approach with their associated definitions.

**Table 4:** *z*_*n*_ Variable definitions.

*z*_*n*_ Variable	Definition
age_45_more	respondent is 45 years or older
aware_po	whether respondents pay attention to palm oil in products or not. Scale: 1 = always to 5 = never
female	1 if respondent is female, 0 if respondent is male
greenvote	1 if respondent is a green party voter, 0 otherwise
children	1 if respondent has children, 0 otherwise
high_education	1 if respondent has university degree or master craftsman, 0 otherwise
inc_abv_av	1 if respondent has an above-average income
consinfluence	whether participants believe that reduction of environmental damage can be induced by consumer behavior and consumption choices. Scale: 1 = ’not convinced at all’ to 5 = ’completely convinced’
pers_sacr	willingness to forgo products to ensure an intact environment. Factor^1^. Scale: 1 = low willingness to make sacrifices to 5 = high willingness to make sacrifices
labeluse	respondents’ frequency of label use. Scale: 1 = ’never’ to 5 = ’always’
trust_organic	whether participants trust the sustainability label or not. Scale: 1 = no top rank to 3 = highest amount of distrust against the label
trust_4-level	whether participants trust the sustainability label or not. Scale: 1 = no top rank to 3 = highest amount of distrust against the label
trust_rspo	whether participants trust the sustainability label or not. Scale: 1 = no top rank to 3 = highest amount of distrust against the label
trust_ngo_sc	whether participants trust organizations such as NGOs or research and science. Factor^1^. Scale: 1 = ’no trust’ to 5 = ’a lot of trust’
trust_off	whether participants trust officials such as authorities, political parties or the media. Factor^1^. Scale: 1 = ’no trust’ to 5 = ’a lot of trust’
trust_impact	whether the participant trusts that labels really have an impact on what they are supposed to signal. Factor^1^. Scale: 1 = ’I do not agree at all’ to ’I fully agree’
concern_futur_env	expresses concern about future development regarding the overall state of the environment. Factor^1^. Scale: respondent 1 = ’doesn’t feel threatened at all’ to 5 = ’feels very threatened’.

^1^: The variables and questions upon which the factor is based can be found in Table A4 in S1 Appendix.

## Empirical results

Table 5 presents the empirical results of the hybrid latent variable analysis for the pooled sample, and both the intervention and non-intervention subsample. We present here a shortened version of the table. The complete table with all parameters of the estimation can be found in Table A5 in S1 Appendix. All estimations are done using the Python package PandasBiogeme. The interested reader is referred to e.g. [65]. The estimates for the choice experiment part of the model are presented in the upper third of Table 5. The second part of the table shows the parameters associated with the ANA part of the estimation, while the third part contains the estimates of the variables that contribute to the definition of the continuous LV, which in turn links the ANA to the choice model part.

**Table 5:** Maximum likelihood estimates pooled sample, information, and no information treatments.

	Pooled Sample			No Information			Information		
**Name**	**Est.**	**Rob. Std err**	**t-test**	**Est.**	**Rob. Std err**	**t-test**	**Est.**	**Rob. Std err**	**t-test**
ASC_NOBUY_LA	0.0157	0.281	0.0557	-0.0969	0.308	-0.314	-0.334	0.246	-1.36
ASC_NOBUY_REF	-0.654***	0.239	-2.74	-1.88***	0.391	-4.8	-1.88***	0.584	-3.21
*λ*_ORGANIC_LA	-0.0658	0.0492	-1.34	-0.496***	0.0965	-5.15	-0.213*	0.122	-1.74
*β*_ORGANIC_REF	1.22***	0.0832	14.6	1.96***	0.186	10.5	2.47***	0.437	5.66
*λ*_BRAND_LA	0.651***	0.17	3.82	-0.213	0.185	-1.15	-0.0455	0.148	-0.308
*β*_BRAND_REF	0.0748**	0.0336	2.23	0.337**	0.137	2.45	0.134	0.0996	1.35
*λ*_4-Level_LA	-0.587***	0.124	-4.73	-0.71***	0.161	-4.4	-1.95*	1.15	-1.69
*β*_4-Level _REF	0.398***	0.067	5.94	0.556***	0.177	3.15	1.01***	0.155	6.53
*λ*_PRICE_LA	0.078	0.159	0.491	-0.186	0.182	-1.02	-0.483***	0.121	-3.99
*β*_PRICE_REF	-0.438***	0.0897	-4.88	-1.21***	0.107	-11.4	-1.12***	0.205	-5.46
*λ*_RSPO_LA	-0.418***	0.157	-2.66	-0.606***	0.135	-4.47	-2.86***	1.03	-2.79
*β*_RSPO_REF	0.324***	0.0711	4.56	0.568***	0.17	3.34	0.838***	0.052	16.1
*β*^*I*^_anaorganic	0.278***	0.075	3.71	0.363***	0.104	3.5	0.28**	0.119	2.35
*β*^*I*^_anabrand	-0.962**	0.395	-2.43	-0.679	0.427	-1.59	-1.12*	0.576	-1.95
*β*^*I*^_ana4-Level	0.309***	0.0944	3.27	0.277***	0.0945	2.93	0.364***	0.137	2.65
*β*^*I*^_anaprice	-0.242***	0.0133	-18.2	-0.466***	0.067	-6.96	-0.255***	0.048	-5.32
*β*^*I*^_anarspo	0.308***	0.0798	3.86	0.282***	0.0887	3.18	0.393***	0.142	2.77
$\beta_{0}^{I}$_anaorganic	-0.974***	0.294	-3.32	-1.1***	0.29	-3.79	-1.2**	0.5	-2.4
$\beta_{0}^{I}$_anabrand	1.32**	0.603	2.19	0.926*	0.532	1.74	1.78*	0.97	1.83
$\beta_{0}^{I}$_ana4-Level	-0.105	0.0727	-1.45	0.00804	0.0835	0.0963	-0.154*	0.0915	-1.69
$\beta_{0}^{I}$_anaprice	0	-	-	0	-	-	0	-	-
$\beta_{0}^{I}$_anarspo	0.189***	0.0388	4.87	0.382***	0.062	6.16	0.151**	0.0626	2.42
ϕ_age_45_more	0.456***	0.0856	5.32	0.0128	0.448	0.0285	0.535***	0.111	4.84
ϕ_aware_po	0.0392***	0.0147	2.66	0.0488**	0.0244	2	0.0195	0.0187	1.05
ϕ_female	-0.133*	0.0704	-1.89	0.0486	0.231	0.21	-0.612***	0.0919	-6.66
ϕ_greenvote	-0.285***	0.0626	-4.55	-0.376**	0.169	-2.23	-0.185**	0.0766	-2.41
ϕ_children	0.632***	0.0914	6.92	0.615***	0.234	2.63	0.677***	0.148	4.58
ϕ_high_education	-0.273***	0.0711	-3.85	-0.667***	0.122	-5.46	-0.019	0.0752	-0.253
ϕ_inc_abv_av	0.052	0.0962	0.54	-0.413	0.276	-1.49	0.105	0.0864	1.21
ϕ_consinfluence	0.0392***	0.0147	2.66	0.0488**	0.0244	2	0.0195	0.0187	1.05
ϕ_pers_sacr	-0.00382	0.0294	-0.13	0.191*	0.11	1.74	0.0043	0.0435	0.0989
ϕ_labeluse	-0.445***	0.0768	-5.79	-0.791***	0.135	-5.86	-0.386***	0.0694	-5.56
ϕ_trust_oranic	0.202**	0.0831	2.43	0.745*	0.394	1.89	0.0368	0.0628	0.586
ϕ_trust_leh	0.104***	0.0286	3.63	0.201***	0.0605	3.32	0.103***	0.0383	2.69
ϕ_trust_ngo_sc	0.0801	0.062	1.29	-0.0821	0.0903	-0.909	0.227**	0.105	2.17
ϕ_trust_off	0.289***	0.0614	4.7	0.58***	0.11	5.26	0.171***	0.0614	2.78
ϕ_trust_rspo	-0.0185	0.0434	-0.426	-0.0685	0.0666	-1.03	-0.0837	0.0588	-1.42
ϕ_trust_impact	0.049	0.0333	1.47	-0.0106	0.079	-0.134	0.216***	0.0452	4.78
ϕ_concern_futur_env	0.0833	0.065	1.28	-0.18	0.208	-0.868	0.113*	0.0659	1.72
*Respondents*	*460*			*229*			*231*		
*Observations*	*2760*			*1374*			*1386*		
*LL(0)*	*-29420*.*69*			*-10942*.*6*			*-10776*.*52*		
*LL*	*-21841*.*6*			*-10942*.*23*			*-10774*.*69*		
*Parameters*	*46*			*46*			*46*		

***, **, * Significance at the 1%, 5%, and 10% levels, respectively. LA: latent attitude.

By means of the associated variables, we can interpret the latent attitude of the respondents along the LV and subsequently its influence on the rest of the model. The signs of the estimates for the LV model suggest that a person with a high LV is more likely to be older than 45, to pay attention to palm oil, to be male, to have children, to have a lower education, to be confident that consumption decisions can have an impact on reducing environmental damage, is less likely to be a green party voter, and to use labels often.

The upper part of the table shows the coefficients of the choice part of the hybrid model. The λ-coefficients describe the influence of the latent variable on the sensitivity to the corresponding attribute. The remaining coefficients are the usual betas of the product attributes. With positive signs for the sustainability-indicating labels and a negative sign for the price attribute as well as for the alternative specific constant (ASC) for the no-buy option, we find the expected signs for all betas. It can be observed that the attribute ‘organic’ shows the largest coefficient, the 4-Level and the RSPO labels are rated quite similarly, and the brand shows the smallest coefficient. All beta coefficients are statistically significant at the 1% level. If we look at the λ-coefficients, we notice that all sustainability-indicating labels show negative signs. Price and brand show positive signs, indicating that individuals in the high range of the LV are more likely to have an increased sensitivity to price and brand and a decreased sensitivity to the sustainability-indicating labels. This implies that with a high LV, the overall coefficient of e.g., RSPO decreases, while the overall coefficient of brand increases. It is interesting to note that the λ-coefficients of organic and price are not statistically significant in the pooled sample. This allows the assumption that the sensitivity directed to these two attributes is not influenced by the latent structure, suggesting that individuals across the range of LV assess these attributes relatively consistently, and are less driven by their own latent attitudes. This finding is consistent with the notion that most people are price-sensitive in their purchases of traditional consumer products, regardless of their personal beliefs.

We next turn to the model part for the responses to the non-attendance or rather ranking questions. With $\beta_{0}^{I}$_anaprice normalized to 0, the $\beta_{0}^{I}$ coefficients indicate a lower initial, stated ANA to the attribute ‘organic’ and a higher initial, stated ANA to ‘brand’ and ‘RSPO’. The signs of the remaining *β*^*I*^ coefficients indicate that as the LV *α*_*nk*_ increases, the probability that respondent *n* ignored the attribute also increases. In other words, the higher the LV *α*_*nk*_, the lower the attendance to all three sustainability-indicating attributes (or small LV value respondents have a higher probability of attending to sustainability labels). And the higher the LV *α*_*nk*_, the greater the attendance to brand and price. These findings are in line with our previous findings regarding the choice model component, since people within the high range of the LV also seem to have a lower sensitivity to the sustainability indicating labels. Thus, the latent variable significantly scales the coefficient of the choice model part, while also influencing consumers’ patterns of attribute non-attendance.

We next consider the results by information intervention group. We hypothesize that factual information influences choice behavior, latent structures, and the use of heuristics. Table 2 shows that individuals in the information intervention group achieved a significantly higher test score. It can thus be hypothesized that the information provided contributed, at least in part, to individuals’ knowledge formation. We can confirm this relationship by presenting the results of an ordered probit model that controls for the explanatory variables used in explaining the LV, as well as the information intervention. The results of the ordered probit are presented in Table A7 in S1 Appendix. Since most of the parameter estimates of the LV approach in Table 5 have the expected signs, we only focus on the major differences between the two subsamples.

We first focus on the LV part of the model comparing the ’No Information’ and ’Information’ columns of Table 5. We find that awareness of palm oil, high education, the belief in the influence of consumption choices and the willingness to make personal sacrifices are not statistically significant. In other words, for participants who received information, these characteristics no longer play a role in the latent structure that influences ANA and individuals’ choice behavior. Using the example of the educational level, it implies that after an information intervention, someone’s initial educational status may lose its influencing power when they are making complex food decisions. On the other hand, information also seems to trigger attitudinal characteristics which in turn interfere with ANA and choice structures. We find that trust in NGOs and science, the belief that consumption choices can have an impact, concern about future development regarding the overall state of the environment, as well as age and gender were statistically significant. These findings suggest that information can help trigger latent trust structures that are then used to resolve complex decision-making processes. Furthermore, information interventions appear to be perceived and processed differently by individuals with different socioeconomic characteristics. For example, we observe that with intervention, age takes on a significant role in forming the LV. We also note that gender is not only statistically significant in the information intervention group, but also shows a reversed sign. This indicates that in the no-intervention group, being female increases the value of LV, while in the intervention group, it decreases the value of LV. This is of particular interest, as women have a significantly higher knowledge score after the information intervention than men (Table A8 in S1 Appendix), suggesting that women may have read the information more carefully or processed it more effectively, enabling them to incorporate the information into their decision-making processes.

Turning to the ANA part of the model, the results show that the coefficient for ANA-organic is smaller for individuals in the information intervention group. Also, the value of the negative ANA-brand coefficient is larger in this group. The value of the negative ANA-price coefficient is smaller in the intervention group. These results indicate that, when consumers have information, the probability that they will consider organic and brand compared to when they do not have information increases, if the LV increases. Furthermore, the probability that price receives less attention in the information intervention group also increases. However, in the choice model part, we find that the λ-coefficient for brand is not statistically significant in both subsamples. In other words, while there are differences in the attention paid to the brand between the groups, the importance of the brand is generally not rated highly (or is only rated so by a small subset of people within the distribution of the LV) as that of other product attributes. In contrast to the non-information group, we find a significant λ-coefficient for the price, suggesting that individuals with different values of LV now evaluate the price differently. In particular, individuals with smaller LV now tend to have a smaller price coefficient, and there is no longer any consensus on the valuation of the price. We find a reverse pattern for the attribute ‘brand’. Since the coefficients of the choice model part have the same expected signs in both groups, we now turn to the WTP estimates in the next section to better illustrate the intuition of the importance of the estimated coefficients using monetary values.

### Willingness-to-pay estimates

This section presents the monetary valuations calculated for the four attributes in Tables 6–9. The tables feature the mean WTP and the bounds at 95% confidence intervals (CI) for the full sample and both subsamples. Furthermore, we present the WTP estimates of the first and the fourth quartile of the predicted LV *α*_*nk*_. As in Bello and Abdulai [16], the means and 95% CIs represent empirical distributions computed using the parametric bootstrap procedure [66] and based on 10,000 replications.

**Table 6:** Average willingness-to-pay estimates.

	Full Sample			No Information Treatment			Information Treatment		
Attribute	WTP	CI		WTP	CI		WTP	CI	
Organic	2.66	[2.09,	5.40]	1.38	[1.01,	2.19]	3.27	[2.39,	4.3]
Brand	0.27	[0.04,	0.51]	0.27	[0.08,	0.63]	0.24	[-0.01,	0.61]
4-Level	0.94	[0.55,	2.44]	0.39	[0.19,	0.73]	0.62	[0.33,	21.4]
RSPO	0.72	[0.38,	1.97]	0.40	[0.20,	0.74]	1.49	[0.36,	78.4]

Note: 95% confidence intervals (CI) are based on 10,000 replications.

**Table 7:** Willingness-to-pay estimates first and fourth quartile of predicted LV, full sample.

	First Quartile (0.00–0.25)			Fourth Quartile (0.75–1.00)		
Attribute	WTP	CI		WTP	CI	
Organic	2.97	[2.02,	7.79]	2.37	[2.03,	3.54]
Brand	0.16	[0.02,	0.38]	0.41	[0.08,	0.69]
4-Level	1.50	[0.77,	4.72]	0.53	[0.35,	0.94]
RSPO	1.03	[0.48,	3.55]	0.47	[0.28,	0.89]

Note: 95% confidence intervals (CI) are based on 10,000 replications.

**Table 8:** Willingness-to-pay estimates first and fourth quartile of predicted LV, no information treatment.

	First Quartile (0.00–0.25)			Fourth Quartile (0.75–1.00)		
Attribute	WTP	CI		WTP	CI	
Organic	1.96	[1.42,	3.52]	0.86	[0.52,	1.55]
Brand	0.28	[0.09,	0.71]	0.26	[0.07,	0.70]
4-Level	0.68	[0.35,	1.29]	0.17	[0.05,	0.43]
RSPO	0.62	[0.31,	1.27]	0.21	[0.09,	0.42]

Note: 95% confidence intervals (CI) are based on 10,000 replications.

**Table 9:** Willingness-to-pay estimates first and fourth quartile of predicted LV, information treatment.

	First Quartile (0.00–0.25)			Fourth Quartile (0.75–1.00)		
Attribute	WTP	CI		WTP	CI	
Organic	2.59	[2.06,	3.23]	4.01	[2.63,	5.59]
Brand	0.16	[-0.01,	0.36]	0.33	[-0.01,	0.92]
4-Level	1.47	[0.68,	70.6]	0.13	[0.07,	3.26]
RSPO	4.59	[0.84,	302]	0.09	[0.04,	0.49]

Note: 95% confidence intervals (CI) are based on 10,000 replications.

Table 6 presents the average WTP estimates for the full sample and subsamples. From the results, it is evident that the attribute ‘organic’ receives the highest monetary valuation in the full sample as well as in both subsamples. The estimates also reveal that with the exception of the brand, the WTP for the attributes are generally higher for the subsample that obtained information, compared to the no-information treatment. The WTP for the attribute ‘brand’ appears to be similar across the full sample and subsamples. It is evident from the estimates that organic certification attracts the highest WTP among the respondents. Interestingly, the rating of the 4-Level label in the overall sample is higher than that of the RSPO label. In the subsample without information, the monetary valuation is virtually the same, suggesting that individuals rate the RSPO label almost the same, if not lower than a hypothetical label that they have only experienced within an experiment. This fact allows initial conclusions to be drawn about the significance of the RSPO label from the consumer’s point of view.

Because consumers have heterogeneous preferences that are not attributable to the treatment groups, it is important to capture this heterogeneity [67]. The latent variable captures a changing structure among respondents, allowing us to use it to consider and reveal those heterogeneous preference and WTP structures. We thereby do not assume latent classes with class-specific, homogeneous preferences, but are able to estimate the WTP for any conceivable manifestation of the latent structure. Table 7 shows the WTP estimates of the first and fourth quartile of the predicted LV *α*_*nk*_ for the full sample to control for differences in the WTP along the LV. We consider the upper and lower quartiles to illustrate extreme values of *α*_*nk*_.

The results in Table 7 reveal that individuals with a low LV tend to exhibit a higher monetary appreciation for all sustainability-indicating labels than individuals with high LV values. However, an individual with high LV is more likely to attribute a stronger WTP with respect to brand. The difference for ‘organic’ is only 20%, but the WTP for the other three attributes varies substantially. This observation already raises the question of whether the attribute ‘organic’ is given a more stable rating across the LV than the other product attributes.

Looking at the WTP in the first and fourth quartiles of predicted LV provides deeper insights into the effect of the information intervention. Tables 8 and 9 reveal the same pattern as before for both groups. As the LV increases, the WTP for the sustainability-indicating labels decreases, and the WTP for brand increases. However, it becomes evident that in the non-intervention group the brand is evaluated rather stably across the LV. Based on these findings, it can be assumed that, without further information, individuals of all LV values consider or evaluate the brand similarly strongly (or weakly) in their product choice processes.

The estimates in Table 9 reveal a higher monetary rating for ‘organic’ and ‘brand’ attributes in the fourth quartile than in the first quartile, while the other sustainability indicators play almost no role in the fourth quartile with information treatment. It should be noted that the WTP value of the RSPO attribute in the first quartile of Table 9 is not interpretable. Individuals in the first quartile of the LV reveal increased monetary appreciation of all sustainability indicators and significantly reduced monetary appreciation for the brand. These findings suggest that the attribute ‘organic’ has the potential to be ranked highest across the entire latent structure. First, we only observe a minor difference between the RSPO and the 4-Level label, and second, there is no clear consensus along consumers’ LV on how to rate these labels monetarily. In contrast to Hinkes and Christoph-Schulz (2020) whose estimates show a decreasing WTP for the EU organic label, our estimates show that the provision of information generates higher WTP among consumers, especially for the organic label. However, it can at the same time contribute to uncertainty, which is reflected in the differences in the monetary valuation of the labels throughout the LV landscape.

To account for differences in the evaluation of different products, we also performed the analyses presented here for separate product groups. The results from the analyses are presented in Tables A5, A9-A11 in S1 Appendix. The estimates reveal comparable WTP patterns across product groups. However, for chocolate spread, brand seems to play a bigger role. At the same time, the RSPO label plays a rather negative role in product evaluation.

## Conclusion

It is widely documented in the empirical literature that consumers face uncertainties in making product choices. Several studies have therefore shown that providing additional information that help consumers in their decision-making process contribute to reducing the bias in discrete choice experiments. In this study, we examined consumer preferences and willingness-to-pay (WTP) for certified palm oil, as well as the effect of information on consumers’ food choice behavior and patterns of attribute non-attendance (ANA). We used a hybrid latent variable scaling approach to model consumer preferences, together with attribute processing-strategies in the context of palm oil-containing food products [16]. In particular, we addressed which sustainability label consumers pay most attention to, and which label at the same time elicits the greatest WTP. This can be particularly important for manufacturing companies and listing retailers, as they can selectively focus on and invest in the certification that consumers prefer. The empirical results revealed increased WTP for sustainability-indicating product attributes, a finding that is consistent with results from previous studies [6, 9, 16].

Our results also showed that providing additional information tends to change consumers’ non-attendance patterns as well as WTP values. In particular, the information intervention tends to increase consumers’ WTP and reduces their ANA for sustainability-indicating attributes. Our estimates revealed that as information is provided, WTP tends to move toward sustainability indicators and away from other product attributes [10]. Our findings suggest that consumers of the various segments analyzed consistently showed the highest WTP values for the attribute ‘organic quality’, indicating that organic quality is one of the most important product attributes from the consumer’s perspective. This is an important finding, as this specific product quality has had a very small market share to date. This finding also raises the question of whether producers should use the EU or German organic label when indicating organic quality.

We also found that consumer valuation of product attributes tends to vary widely across the sample, particularly within the information treatment subsample. Specifically, while with information provision some consumers have almost no WTP at all for brand, the WTP increases for other consumers. Our results further revealed that a hypothetical label that consumers are familiar with only by design, but not within the palm oil context, also exerts a positive influence on the valuation by a particular consumer group, resulting in increased WTP. However, this positive influence was not observed across other consumer segments, which contrasts with the findings for organic certified palm oil, which showed higher WTP across all consumer segments. This finding indicates that organic certified palm oil is more suitable as a sustainability indicator for influencing consumers’ purchasing decisions. The findings also underscore the significance of providing factual information, as this increases the willingness to buy more sustainable products and at the same time shifts consumers’ focus (ANA structures) away from price and towards sustainability indicators. However, there is no guarantee that an organic certified product is in fact a more sustainable product, and organic certification appears to be the more important consideration purely from the point of view of German consumers.

Future research should examine this question more closely with regard to ecological and possibly social impacts. Our findings suggest that in order to set the palm oil sector on a more sustainable path, stakeholders should focus on sustainability labels that have already been accepted and valued by consumers. The organic label particularly appears to be important in this context. To the extent that some consumers value brands, producers could make use of brand names to attract some consumer groups. The RSPO which was founded to promote sustainable agricultural practices in the palm oil sector appears to play a relatively negligible role for consumers. Nevertheless, the importance of the RSPO label should not be underestimated. For producers, it is currently one of the few labels to indicate sustainability within their supply chain and represents a way to enhance a gradual shift towards more sustainable production methods. In line with recent literature, it is evident that consumers prefer segregated supply chains. Gassler and Spiller [9] also indicate that consumers expect at least a guaranteed minimum of sustainable palm oil in a product if it carries a label. However, in their study they were only able to evaluate the RSPO label, which does not seem to receive wide acceptance from a consumer perspective. Implications based on this approach could lead to misleading conclusions, as the label in itself is accorded low importance. However, consistent with their findings, we find relatively similar WTP values for RSPO certification. Also, in line with findings by Hinkes and Christoph-Schulz [10], we observe negative WTP values for the RSPO certification in some consumer groups. Our analysis thus helps shed more light on consumers’ perceptions of these labels. From the consumer’s point of view, oil palm cultivation systems should be converted to organic agriculture in the long run. At the same time, however, the indicator system could also be redesigned to give a more consistent look throughout all product categories, which would make it much easier for consumers to evaluate products of all kinds. Future research could also explore the extent to which a simple label design can holistically promote sustainability, in a simplified way, and for different product groups. It is significant to note that our results can only be interpreted on the basis of our survey sample. Although this sample broadly reflects the German populace as a whole in terms of various characteristics, it is not representative. At the same time, we cannot be sure to what extent the treatment group actually read the information carefully. Furthermore, it is possible to provide information in such a visual and educational way that it has a greater educational effect. In order to develop highly effective information campaigns, it is essential that these aspects receive further attention. Our findings also suggest that producers are willing to use more organic certified palm oil if the market allows it suggesting that consumers and producers are not too far apart in their market and product expectations. However, there seems to be a market reluctance, among producers for example, to establish their own organic plantations or sustainable projects. We also suggest that it seems important that producers are given more information about the oil they use, as it appears that issues around transparency are already occurring in the wholesale sector. More information about the other side of the market–that is, improved information exchange and communication–could thus move the markets in a direction that would be more consistent with the expectations of both consumers and producers.

## Supporting information

S1 Appendix (DOCX)

S1 Data Data format for descriptive statistics. (DTA)

S2 Data Data format for choice model analysis. (DAT)

S3 Data Data palm oil using companies in Germany. (DTA)
