# Supplementary material for: The role of information in consumer preferences for sustainable certified palm oil products in Germany
Source: PLoS One. 2022 Jul 25;17(7):e0271198. doi: 10.1371/journal.pone.0271198 (PMC9312394; doi:10.1371/journal.pone.0271198)
Supplement: S1 Appendix — (DOCX) [file pone.0271198.s001.docx]

# **S1 Appendix**

## **I. Information Intervention**

| **Table A1. Company Sizes** | |
| --- | --- |
| Company size  (number of employees) | Percent |
| 1 – 19 | 9.10 |
| 20 – 49 | 11.49 |
| 50 – 99 | 5.75 |
| 100 – 249 | 20.69 |
| 250 – 499 | 16.09 |
| > 500 | 36.78 |
| n = 87 | 100.00 |

## **II. Information Intervention**

Some information presented here has changed in the meantime. To the best of our knowledge, all information was accurate at the time of the survey.

*The following questionnaire will mainly discuss the topic of sustainable consumption within the context of palm oil as an agricultural commodity. Therefore, we would like to give you some general information on the topic of oil palm and palm oil cultivation.*

1. Palm oil is a vegetable oil that is used as an ingredient in food, cosmetics and also as biodiesel. Since 2014, it must be clearly labeled as an ingredient on food products.
2. Statistically, palm oil is currently present as an ingredient in one out of every two food products available in supermarkets.
3. Oil palms, like coconut palms, grow exclusively in the warm, humid tropics. They need high temperatures and rainfall all year round to thrive.
4. Palm oil is the most produced, traded and consumed vegetable oil worldwide. Through employment opportunities in cultivation or the development of infrastructure in growing regions, the oil palm offers many people a way to earn their livelihood while the regions can develop economically.
5. At the same time, palm oil cultivation on large plantations is also partly responsible for the displacement of indigenous populations from their lands in cultivation areas, either because property rights are not clearly regulated or because large corporations grab lands without obtaining permits or paying compensation. Such actions are sometimes made possible by corruption in cultivating countries. In the event that the originally resident population has to access new areas for agricultural use, the destruction of forest areas is thus further promoted.
6. The European Union is the second largest importer of palm oil after India.
7. The oil palm is the most productive oil plant in the world. Worldwide, the average yield per hectare is around 3.5 tons. Other oil plants, such as soy or rapeseed, however, only manage yields of 0.5 to 1.8 tons per hectare. This means that (compared to other plants) a lot of oil can be produced with a relatively low land consumption. For example, replacing palm oil with sunflower oil would require about four times more land to produce the same amount of oil.
8. The world's largest share of palm oil is produced on large monoculture plantations in Indonesia and Malaysia. The clearing of primary rainforest areas leads to a decline in animal populations and to the loss of habitats for wild animals. A famous example is the orangutan. The loss of forests and peat bogs, which in contrast to the established plantations have an increased CO2 storage potential, also has a direct impact on the global CO2 balance and thus on man-made climate change. In order to gain new areas, slash-and-burn clearings are often practiced.
9. Currently, about 38% less land is used for palm oil cultivation than for rapeseed cultivation.

## **III. Basic Information on labels and certifications used in the Choice Experiment**

**The products available for selection can carry three different labels** (the labels always refer to the ingredient palm oil)**:**

| **Table A2. Basic Information** | | |
| --- | --- | --- |
| **1.** | **German Bio** (organic) **Label** | 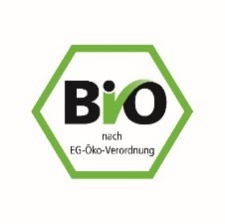 |
|  | The Federal Ministry of Food and Agriculture grants the German organic label. It is therefore a label with state-controlled requirements. | |
| **2.** | **RSPO Label** | 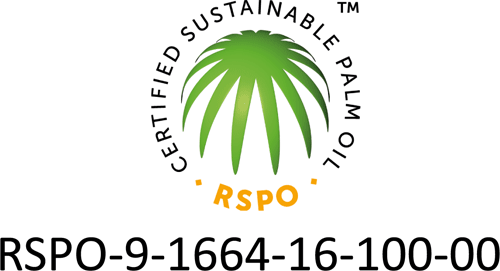 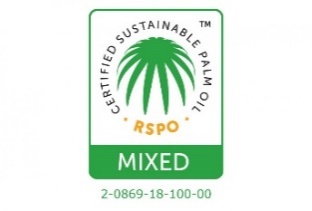 |
|  | The Roundtable on Sustainable Palm Oil was founded on the initiative of WWF and Unilever. Members of the Roundtable are environmental protection associations, non-governmental organizations, corporations, banks and other institutions involved in the production and processing of palm oil. It is therefore a label with a private character.  MIXED (or Mass Balance) means that there is partly RSPO certified and partly non-certified palm oil in the product. The exact proportions are not known. | |
| **3.** | **4-Level Label** | 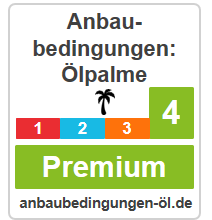 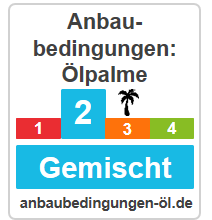 |
|  | The 4-level label refers to the cultivation conditions of the oil palm. As with the new animal husbandry label, the label shown here is established by retailers (e.g. Rewe, Lidl, Aldi, Edeka, etc.). The retailers are thus also responsible for the definition of the criteria, as well as the organization of the control. It marks a gradual improvement in individual social and ecological aspects through the stages. In the context of granting the label, all sustainability aspects (such as an overall footprint) of the respective product are included in the evaluation. In the experiment, you will find level 4 (Premium) or 2 (similar to the MIXED/ Mass Balance as described before). | |

## **IV. Choice Set Example**

**Table A3. Choice Set 16**

| **Attribute** | **Alternative 1** | **Alternative 2** | **Opt-Out** |
| --- | --- | --- | --- |
| Brand | other product | well-known brand product | None of the products |
| Organic | - | 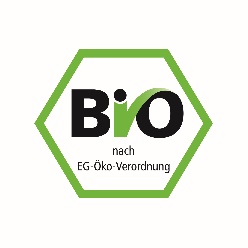 |  |
| RSPO | - | 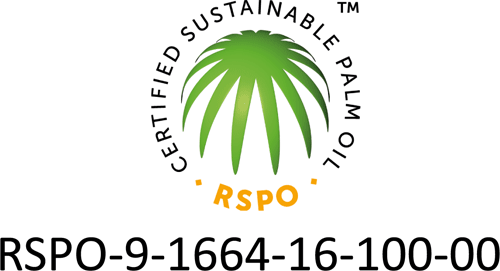 |  |
| 4-Level | 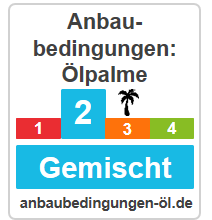 | 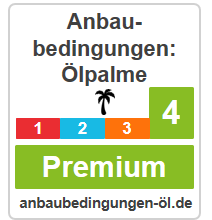 |  |
| Price | 0.99 | 1.29 |  |
| I choose: |  |  |  |

## **V. Cheap talk Script**

After a general introduction to the experiment, we presented a cheap talk script directly before the first decision-making situation. In the following, we first provide an analogous translation and then the original German version.

“Previous experiments of this kind have shown that people often choose products that they would not choose in a real shopping situation. One reason for this behavior is that while they would like to buy the product, in reality they are not willing to pay the quoted price. Therefore, please think about which characteristics you personally value and to what extent your available budget for food affects your decision.”

„Vorangegangene Experimente dieser Art haben gezeigt, dass Menschen oftmals Produkte auswählen, für die sie sich in der Realität jedoch nicht entscheiden würden, da sie das Produkt zwar gerne kaufen möchten, in der Wirklichkeit jedoch gar nicht bereit sind, den angegebenen Preis zu zahlen. Überlegen Sie sich deshalb bitte, auf welche Eigenschaften Sie persönlich Wert legen und inwieweit Ihr zur Verfügung stehendes Budget für Lebensmittel Ihre Entscheidung beeinflusst.“

## **VI. Knowledge Quiz: Statements to agree or disagree with**

1. Due to the endangered tropical cultivation regions, producers should increase the use of coconut oil instead of palm oil as an ingredient in food to prevent further environmental damage.

2. Due to its efficient yield per unit area, oil palm has the potential to be a relatively sustainable oil crop.

3. In order to improve the living conditions of people in palm oil cultivation areas, the EU should impose a general ban on palm oil.

4. Palm oil is found in relatively few products, so it is not a major concern when choosing a food product.

5. Current oil palm cultivation practices are largely responsible for CO_2_ emissions and biodiversity loss in Southeast Asia.

6. It is often not possible for consumers to identify palm oil as an ingredient in food products.

## **VII. Price expectation Questions**

A) (Forward Looking Approach)

What price would you expect to pay for a [*standard unit*] of your favorite [*product*] in the supermarket during your next purchase? ________ €

I expect the price of my favorite [*product*] not to be HIGHER than ________€ in 90% of stores.

I expect the price of my favorite [*product*] not to be LOWER than ________€ in 90% of stores.

B) (Backward Looking Approach)

What price did you pay for a [*standard unit*] of your favorite [*product*] at the supermarket the last time you bought it? ________ €

I think that the price of my favorite [*product*] at the time of my last purchase was not HIGHER than ________€ in 90% of stores.

I think that the price of my favorite [*product*] at the time of my last purchase was not LOWER than ________€ in 90% of stores.

## **VIII. Factor variables**

| **Table A4. Factor Variables** | | |
| --- | --- | --- |
| pers_sacr | 1. | In order to preserve our natural livelihoods, I would be willing to completely abandon tropical food products from my diet. |
|  | 2. | In order to preserve our natural livelihoods, I would be willing to completely abandon animal-based food products from my diet. |
| Which people, groups or organizations do you trust more or less?  How much trust do you place in... | | |
| trust_NGO_sc | 1. | … organizations (e.g. consumer or environmental organizations, other NGOs)? |
|  | 2. | … independent research institutes? |
| trust_off | 1. | … in media? |
|  | 2. | … authorities? |
|  | 3. | … political parties? |
|  | 4. | … large commercial enterprises? |
| trust_impact | 1. | One can trust in the validity of labels on food products in German supermarkets, as producers and manufacturers are controlled in an effective way. |
|  | 2. | If a product carries a certain certification, then I don't question that assurance. |
| concern_futur_env | 1. | Please indicate how threatened you feel by the following issues in terms of preserving our natural livelihoods: Climate change. |
|  | 2. | It worries me to think of the environmental conditions our children and grandchildren are likely to have to live in. |
|  | 3. | Please indicate how threatened you feel by the following issues in terms of preserving our natural livelihoods: Consumption of food of animal origin in the future. |

## **IX. Complete Result Tables**

| **Table A5. Maximum Likelihood Estimates Pooled Sample, Information, and No Information Treatments, Complete Estimation Table** | | | | | | | | | | | | |
| --- | --- | --- | --- | --- | --- | --- | --- | --- | --- | --- | --- | --- |
|  | **Pooled Sample** | | |  | **No Information** | | |  | **Information** | | | |
| **Name** | **Est.** | **Rob. Std err** | **t-test** |  | **Est.** | **Rob. Std err** | **t-test** |  | **Est.** | **Rob. Std err** | **t-test** | |
| ASC_NOBUY_LA | 0.0157 | 0.281 | 0.0557 |  | -0.0969 | 0.308 | -0.314 |  | -0.334 | 0.246 | -1.36 | |
| ASC_NOBUY_REF | -0.654*** | 0.239 | -2.74 |  | -1.88*** | 0.391 | -4.8 |  | -1.88*** | 0.584 | -3.21 | |
| $\lambda\_$ORGANIC_LA | -0.0658 | 0.0492 | -1.34 |  | -0.496*** | 0.0965 | -5.15 |  | -0.213* | 0.122 | -1.74 | |
| $\beta$_ORGANIC_REF | 1.22*** | 0.0832 | 14.6 |  | 1.96*** | 0.186 | 10.5 |  | 2.47*** | 0.437 | 5.66 | |
| $\lambda\_$BRAND_LA | 0.651*** | 0.17 | 3.82 |  | -0.213 | 0.185 | -1.15 |  | -0.0455 | 0.148 | -0.308 | |
| $\beta$_BRAND_REF | 0.0748** | 0.0336 | 2.23 |  | 0.337** | 0.137 | 2.45 |  | 0.134 | 0.0996 | 1.35 | |
| $\lambda\_$LEH_LA | -0.587*** | 0.124 | -4.73 |  | -0.71*** | 0.161 | -4.4 |  | -1.95* | 1.15 | -1.69 | |
| $\beta$_LEH_REF | 0.398*** | 0.067 | 5.94 |  | 0.556*** | 0.177 | 3.15 |  | 1.01*** | 0.155 | 6.53 | |
| $\lambda\_$PRICE_LA | 0.078 | 0.159 | 0.491 |  | -0.186 | 0.182 | -1.02 |  | -0.483*** | 0.121 | -3.99 | |
| $\beta$_PRICE_REF | -0.438*** | 0.0897 | -4.88 |  | -1.21*** | 0.107 | -11.4 |  | -1.12*** | 0.205 | -5.46 | |
| $\lambda\_$RSPO_LA | -0.418*** | 0.157 | -2.66 |  | -0.606*** | 0.135 | -4.47 |  | -2.86*** | 1.03 | -2.79 | |
| $\beta$_RSPO_REF | 0.324*** | 0.0711 | 4.56 |  | 0.568*** | 0.17 | 3.34 |  | 0.838*** | 0.052 | 16.1 | |
| $\beta^{I}$_anaorganic_F1 | 0.278*** | 0.075 | 3.71 |  | 0.363*** | 0.104 | 3.5 |  | 0.28** | 0.119 | 2.35 | |
| $\beta^{I}$_anabrand_F1 | -0.962** | 0.395 | -2.43 |  | -0.679 | 0.427 | -1.59 |  | -1.12* | 0.576 | -1.95 | |
| $\beta^{I}$_analeh_F1 | 0.309*** | 0.0944 | 3.27 |  | 0.277*** | 0.0945 | 2.93 |  | 0.364*** | 0.137 | 2.65 | |
| $\beta^{I}$_anaprice_F1 | -0.242*** | 0.0133 | -18.2 |  | -0.466*** | 0.067 | -6.96 |  | -0.255*** | 0.048 | -5.32 | |
| $\beta^{I}$_anarspo_F1 | 0.308*** | 0.0798 | 3.86 |  | 0.282*** | 0.0887 | 3.18 |  | 0.393*** | 0.142 | 2.77 | |
| $\beta_{0}^{I}$_anaorganic | -0.974*** | 0.294 | -3.32 |  | -1.1*** | 0.29 | -3.79 |  | -1.2** | 0.5 | -2.4 | |
| $\beta_{0}^{I}$_anabrand | 1.32** | 0.603 | 2.19 |  | 0.926* | 0.532 | 1.74 |  | 1.78* | 0.97 | 1.83 | |
| $\beta_{0}^{I}$_analeh | -0.105 | 0.0727 | -1.45 |  | 0.00804 | 0.0835 | 0.0963 |  | -0.154* | 0.0915 | -1.69 | |
| $\beta_{0}^{I}$_anaprice | 0 | - | - |  | 0 | - | - |  | 0 | - | - | |
| $\beta_{0}^{I}$_anarspo | 0.189*** | 0.0388 | 4.87 |  | 0.382*** | 0.062 | 6.16 |  | 0.151** | 0.0626 | 2.42 | |
| $\sigma$_anabio | 0.757*** | 0.213 | 3.56 |  | 0.984*** | 0.198 | 4.96 |  | 0.819*** | 0.312 | 2.62 | |
| $\sigma$_anabrand | 0.634*** | 0.0554 | 11.4 |  | 1.39*** | 0.17 | 8.13 |  | 0.67*** | 0.0856 | 7.83 | |
| $\sigma$_analeh | 0.589*** | 0.162 | 3.64 |  | 0.795*** | 0.138 | 5.76 |  | 0.668** | 0.266 | 2.51 | |
| $\sigma$_anaprice | 0.91*** | 0.281 | 3.24 |  | 0.977*** | 0.328 | 2.98 |  | 1.07** | 0.441 | 2.42 | |
| $\sigma$_anarspo | 0.597*** | 0.176 | 3.4 |  | 0.717*** | 0.128 | 5.59 |  | 0.746** | 0.303 | 2.46 | |
| ϕ_age_45_more | 0.456*** | 0.0856 | 5.32 |  | 0.0128 | 0.448 | 0.0285 |  | 0.535*** | 0.111 | 4.84 | |
| ϕ_awarep | 0.0392*** | 0.0147 | 2.66 |  | 0.0488** | 0.0244 | 2 |  | 0.0195 | 0.0187 | 1.05 | |
| ϕ_female | -0.133* | 0.0704 | -1.89 |  | 0.0486 | 0.231 | 0.21 |  | -0.612*** | 0.0919 | -6.66 | |
| ϕ_greenvote | -0.285*** | 0.0626 | -4.55 |  | -0.376** | 0.169 | -2.23 |  | -0.185** | 0.0766 | -2.41 | |
| ϕ_haveChildren | 0.632*** | 0.0914 | 6.92 |  | 0.615*** | 0.234 | 2.63 |  | 0.677*** | 0.148 | 4.58 | |
| ϕ_highEducation | -0.273*** | 0.0711 | -3.85 |  | -0.667*** | 0.122 | -5.46 |  | -0.019 | 0.0752 | -0.253 | |
| ϕ_incMoreAv | 0.052 | 0.0962 | 0.54 |  | -0.413 | 0.276 | -1.49 |  | 0.105 | 0.0864 | 1.21 | |
| ϕ_consinfluence | 0.0392*** | 0.0147 | 2.66 |  | 0.0488** | 0.0244 | 2 |  | 0.0195 | 0.0187 | 1.05 | |
| ϕ_pers_sacr | -0.00382 | 0.0294 | -0.13 |  | 0.191* | 0.11 | 1.74 |  | 0.0043 | 0.0435 | 0.0989 | |
| ϕ_labeluse | -0.445*** | 0.0768 | -5.79 |  | -0.791*** | 0.135 | -5.86 |  | -0.386*** | 0.0694 | -5.56 | |
| ϕ_trust_oranic | 0.202** | 0.0831 | 2.43 |  | 0.745* | 0.394 | 1.89 |  | 0.0368 | 0.0628 | 0.586 | |
| ϕ_trust_leh | 0.104*** | 0.0286 | 3.63 |  | 0.201*** | 0.0605 | 3.32 |  | 0.103*** | 0.0383 | 2.69 | |
| ϕ_trust_ngo_sc | 0.0801 | 0.062 | 1.29 |  | -0.0821 | 0.0903 | -0.909 |  | 0.227** | 0.105 | 2.17 | |
| ϕ_trust_off | 0.289*** | 0.0614 | 4.7 |  | 0.58*** | 0.11 | 5.26 |  | 0.171*** | 0.0614 | 2.78 | |
| ϕ_trust_rspo | -0.0185 | 0.0434 | -0.426 |  | -0.0685 | 0.0666 | -1.03 |  | -0.0837 | 0.0588 | -1.42 | |
| ϕ_trust_impact | 0.049 | 0.0333 | 1.47 |  | -0.0106 | 0.079 | -0.134 |  | 0.216*** | 0.0452 | 4.78 | |
| ϕ_futur_env | 0.0833 | 0.065 | 1.28 |  | -0.18 | 0.208 | -0.868 |  | 0.113* | 0.0659 | 1.72 | |
| delta_1 | 0.26*** | 0.0706 | 3.68 |  | 0.323*** | 0.0607 | 5.32 |  | 0.314*** | 0.12 | 2.62 | |
| delta_2 | 0.583*** | 0.16 | 3.65 |  | 0.733*** | 0.139 | 5.28 |  | 0.689*** | 0.265 | 2.6 | |
| *Respondents* | *460* |  |  |  | *229* |  |  |  | *231* |  | |  |
| *Observations* | *2760* |  |  |  | *1374* |  |  |  | *1386* |  | |  |
| *LL(0)* | *-29420.69* |  |  |  | *-10942.6* |  |  |  | *-10776.52* |  | |  |
| *LL* | *-21841.6* |  |  |  | *-10942.23* |  |  |  | *-10774.69* |  | |  |
| *Parameters* | *46* |  |  |  | *46* |  |  |  | *46* |  | |  |
| ****, **, * Significance at the 1%, 5%, and 10% levels, respectively.*  *LA = latent attitude* | | | | | | | | | | | | |

| **Table A6. Maximum Likelihood Estimates by Product** | | | | | | | | |
| --- | --- | --- | --- | --- | --- | --- | --- | --- |
|  | **Cookies** | | |  | **Chocolate Spread** | | |  |
| **Name** | **Est.** | **Std. err** | **t-test** |  | **Est.** | **Std. err** | **t-test** |  |
| ASC_NOBUY_LA | -0.317 | 0.203 | -1.57 |  | 0.747** | 0.304 | 2.46 |  |
| ASC_NOBUY_REF | -2.38*** | 0.577 | -4.13 |  | -1.08** | 0.35 | -3.07 |  |
| $\lambda\_$ORGANIC_LA | -0.15 | 0.102 | -1.47 |  | -0.988*** | 0.134 | -7.36 |  |
| $\beta$_ORGANIC_REF | 1.78*** | 0.238 | 7.48 |  | 4.31*** | 0.399 | 10.8 |  |
| $\lambda\_$BRAND_LA | -0.0806 | 0.281 | -0.287 |  | -0.651*** | 0.204 | -3.2 |  |
| $\beta$_BRAND_REF | 0.0747 | 0.102 | 0.731 |  | 0.427** | 0.21 | 2.04 |  |
| $\lambda\_$LEH_LA | -1.2*** | 0.275 | -4.38 |  | -2.64*** | 0.332 | -7.96 |  |
| $\beta$_LEH_REF | 1.07*** | 0.142 | 7.59 |  | 0.235*** | 0.0101 | 23.4 |  |
| $\lambda\_$PRICE_LA | -0.295*** | 0.0875 | -3.37 |  | -0.151 | 0.175 | -0.861 |  |
| $\beta$_PRICE_REF | -1.39*** | 0.163 | -8.54 |  | -1.07*** | 0.142 | -7.52 |  |
| $\lambda\_$RSPO_LA | -1.15*** | 0.363 | -3.18 |  | -2.61*** | 0.345 | -7.56 |  |
| $\beta$_RSPO_REF | 0.941*** | 0.214 | 4.4 |  | -0.0475 | 0.0544 | -0.873 |  |
| $\beta^{I}$_anaorganic_F1 | 0.215*** | 0.0404 | 5.31 |  | 0.334** | 0.156 | 2.15 |  |
| $\beta^{I}$_anabrand_F1 | -0.983*** | 0.245 | -4.02 |  | -0.06 | 0.0813 | -0.738 |  |
| $\beta^{I}$_analeh_F1 | 0.426*** | 0.0865 | 4.93 |  | 0.282** | 0.135 | 2.08 |  |
| $\beta^{I}$_anaprice_F1 | -0.32*** | 0.0309 | -10.3 |  | -0.884** | 0.408 | -2.17 |  |
| $\beta^{I}$_anarspo_F1 | 0.313*** | 0.0534 | 5.86 |  | 0.321** | 0.147 | 2.19 |  |
| $\beta_{0}^{I}$_anaorganic | -1.03*** | 0.18 | -5.74 |  | -1.11** | 0.504 | -2.2 |  |
| $\beta_{0}^{I}$_anabrand | 1.67*** | 0.471 | 3.54 |  | 0.503** | 0.239 | 2.1 |  |
| $\beta_{0}^{I}$_analeh | -0.312*** | 0.109 | -2.88 |  | 0.16** | 0.0763 | 2.1 |  |
| $\beta_{0}^{I}$_anaprice | 0 | - | - |  | 0 | - | - |  |
| $\beta_{0}^{I}$_anarspo | 0.256*** | 0.0507 | 5.04 |  | 0.465** | 0.215 | 2.16 |  |
| $\sigma$_anabio | 0.931*** | 0.156 | 5.96 |  | 1.02** | 0.465 | 2.18 |  |
| $\sigma$_anabrand | 0.846*** | 0.0777 | 10.9 |  | 1.82** | 0.833 | 2.19 |  |
| $\sigma$_analeh | 0.746*** | 0.118 | 6.32 |  | 0.715** | 0.325 | 2.2 |  |
| $\sigma$_anaprice | 1.16*** | 0.204 | 5.69 |  | 0.322** | 0.163 | 1.97 |  |
| $\sigma$_anarspo | 0.716*** | 0.123 | 5.82 |  | 0.823** | 0.376 | 2.19 |  |
| ϕ_age_45_more | 0.698*** | 0.0882 | 7.91 |  | -0.538*** | 0.0801 | -6.72 |  |
| ϕ_awarep | 0.0861*** | 0.0215 | 4 |  | 0.0494*** | 0.0133 | 3.71 |  |
| ϕ_female | -0.405*** | 0.106 | -3.8 |  | 0.0274 | 0.0713 | 0.385 |  |
| ϕ_greenvote | -0.245** | 0.0985 | -2.49 |  | 0.141** | 0.0686 | 2.06 |  |
| ϕ_haveChildren | 1.22*** | 0.153 | 7.93 |  | 0.122 | 0.0953 | 1.29 |  |
| ϕ_highEducation | -0.317*** | 0.096 | -3.3 |  | 0.0286 | 0.081 | 0.353 |  |
| ϕ_incMoreAv | 0.0399 | 0.0978 | 0.408 |  | -0.211*** | 0.0684 | -3.08 |  |
| ϕ_consinfluence | 0.0861*** | 0.0215 | 4 |  | 0.0494*** | 0.0133 | 3.71 |  |
| ϕ_pers_sacr | -0.0641 | 0.0469 | -1.37 |  | 0.152*** | 0.0499 | 3.04 |  |
| ϕ_labeluse | -0.582*** | 0.0617 | -9.43 |  | -0.383*** | 0.0472 | -8.11 |  |
| ϕ_trust_oranic | 0.0633 | 0.0618 | 1.02 |  | 0.646*** | 0.0628 | 10.3 |  |
| ϕ_trust_leh | 0.0364 | 0.0422 | 0.863 |  | -0.0114 | 0.0517 | -0.221 |  |
| ϕ_trust_ngo_sc | 0.303*** | 0.0777 | 3.9 |  | 0.245*** | 0.0661 | 3.71 |  |
| ϕ_trust_off | 0.498*** | 0.0784 | 6.35 |  | 0.082 | 0.0961 | 0.854 |  |
| ϕ_trust_rspo | -0.169*** | 0.0418 | -4.05 |  | -0.261*** | 0.0401 | -6.52 |  |
| ϕ_trust_impact | -0.0916* | 0.05 | -1.83 |  | 0.0564* | 0.0338 | 1.67 |  |
| ϕ_futur_env | 0.235*** | 0.0677 | 3.47 |  | -0.35*** | 0.0784 | -4.46 |  |
| delta_1 | 0.331*** | 0.053 | 6.25 |  | 0.317** | 0.144 | 2.19 |  |
| delta_2 | 0.718*** | 0.115 | 6.23 |  | 0.734** | 0.334 | 2.2 |  |
| *Respondents* | *249* |  |  |  | *211* |  |  |  |
| *Observations* | *1494* |  |  |  | *1266* |  |  |  |
| *LL(0)* | *-15957.16* |  |  |  | *-13777.73* |  |  |  |
| *LL* | *-11805.01* |  |  |  | *-9887.083* |  |  |  |
| *Parameters* | *46* |  |  |  | *46* |  |  |  |
| ****, **, * Significance at the 1%, 5%, and 10% levels, respectively.*  *LA = latent attitude* | | | | | | | | |

## **X. Quiz Score**

| **Table A7. Ordered Probit: Score** | | |
| --- | --- | --- |
| Variables | score |  |
|  |  |  |
| age | -0.0221*** | (0.00347) |
| children | -0.441*** | (0.157) |
| education | 0.0705** | (0.0336) |
| income | 0.0168 | (0.0254) |
| female | -0.0448 | (0.116) |
| consinfluence | -0.0698 | (0.0546) |
| labeluse | -0.0429 | (0.0638) |
| trust_organic | -0.172 | (0.127) |
| trust_rspo | -0.0299 | (0.0658) |
| trust_4-levels | -0.0924 | (0.0621) |
| awareness_po | 0.0809* | (0.0466) |
| trust_impact | 0.00144 | (0.0665) |
| concern_futur_env | -0.105 | (0.0930) |
| pers_sacr | -0.0376 | (0.0631) |
| trust_off | -0.112 | (0.0994) |
| trust_NGO_sc | 0.193** | (0.0958) |
| information | 0.414*** | (0.103) |
| 1. CDU / CSU | -0.386** | (0.158) |
| 2. SPD | -0.343* | (0.203) |
| 4. AFD | -0.570** | (0.246) |
| 5. FDP | -0.210 | (0.229) |
| 6. DIE LINKE | 0.0817 | (0.197) |
| 7. DIE PARTEI | -0.209 | (0.355) |
| 8. others | -0.400** | (0.186) |
| /cut1 | -3.037*** | (0.704) |
| /cut2 | -1.850*** | (0.696) |
| /cut3 | -1.044 | (0.695) |
| /cut4 | -0.305 | (0.693) |
| /cut5 | 0.372 | (0.693) |
|  |  |  |
| Observations | 460 |  |
| *Standard errors in parentheses; *** p<0.01, ** p<0.05, * p<0.1*  *This table presents the results of an ordered probit model which considers the quiz score as the dependent variable. We examine whether the determinants used in the latent variable model as well as the intervention have an influence on the score, that is, on participants' knowledge.* | | |

| **Table A8. t-test: score by sex and subsample status** | | | | | | | |
| --- | --- | --- | --- | --- | --- | --- | --- |
|  | obs | male mean | female mean | diff | st_err | t-value | Pr(T < t) |
| No Information Intervention | 229 | 3.451 | 3.612 | -0.161 | 0.178 | -0.901 | 0.184 |
| Information Intervention | 231 | 3.821 | 4.252 | -0.431 | 0.178 | -2.419 | 0.008 |

## **XI. Willingness-to-pay estimates by Product**

| **Table A9. Willingness-to-pay Estimates by products** | | | | | | |
| --- | --- | --- | --- | --- | --- | --- |
|  | Cookies | | | Chocolate Spread | | |
| Attribute | WTP | CI | | WTP | CI | |
| Organic | 1.59 | [1.18, | 2.17] | 5.12 | [3.03, | 10.70] |
| Brand | 0.08 | [-0.09, | 0.53] | 0.41 | [0.02, | 0.82] |
| 4-Level | 0.42 | [0.25, | 0.86] | 7.40 | [-1.15, | 49.90] |
| RSPO | 0.36 | [0.18, | 1.02] | -2.06 | [-25.40, | 20.20] |
| *Note: 95% confidence intervals (CI) are based on 10,000 replications.* | | | | | | |

| **Table A10. Willingness-to-pay Estimates first and fourth quartile of predicted LV, Cookie** | | | | | | |
| --- | --- | --- | --- | --- | --- | --- |
|  | First Quartile (0.00 - 0.25) | | | Fourth Quartile (0.75 - 1.00) | | |
| Attribute | WTP | CI | | WTP | CI | |
| Organic | 1.35 | [1.10, | 1.80] | 1.82 | [1.14, | 2.68] |
| Brand | 0.06 | [-0.10, | 0.25] | 0.09 | [-0.10, | 0.90] |
| 4-Level | 0.94 | [0.54, | 2.04] | 0.14 | [0.07, | 0.37] |
| RSPO | 0.79 | [0.41, | 2.46] | 0.13 | [0.05, | 0.53] |
| *Note: 95% confidence intervals (CI) are based on 10,000 replications.* | | | | | | |

| **Table A11. Willingness-to-pay Estimates first and fourth quartile of predicted LV, Chocolate Spread** | | | | | | |
| --- | --- | --- | --- | --- | --- | --- |
|  | First Quartile (0.00 - 0.25) | | | Fourth Quartile (0.75 - 1.00) | | |
| Attribute | WTP | CI | | WTP | CI | |
| Organic | 9.56 | [4.61, | 23.90] | 2.31 | [1.73, | 3.48] |
| Brand | 0.61 | [0.04, | 1.21] | 0.26 | [0.02, | 0.59] |
| 4-Level | 25.90 | [-5.72, | 192] | 0.35 | [0.12, | 0.92] |
| RSPO | -7.75 | [-99.20, | 78.70] | -0.06 | [-0.35, | 0.22] |
| *Note: 95% confidence intervals (CI) are based on 10,000 replications.* | | | | | | |
